# Automated Screening for Social Anxiety, Generalized Anxiety, and Depression From Objective Smartphone-Collected Data: Cross-sectional Study

**DOI:** 10.2196/28918

**Published:** 2021-08-13

**Authors:** Daniel Di Matteo, Kathryn Fotinos, Sachinthya Lokuge, Geneva Mason, Tia Sternat, Martin A Katzman, Jonathan Rose

**Affiliations:** 1 The Edward S Rogers Sr Department of Electrical and Computer Engineering University of Toronto Toronto, ON Canada; 2 START Clinic for Mood and Anxiety Disorders Toronto, ON Canada; 3 Department of Psychology Adler Graduate Professional School Toronto, ON Canada; 4 Department of Psychology Lakehead University Thunder Bay, ON Canada; 5 The Northern Ontario School of Medicine Thunder Bay, ON Canada

**Keywords:** mobile sensing, passive EMA, passive sensing, psychiatric assessment, mood and anxiety disorders, mobile apps, mhealth, mobile phone, digital health, digital phenotyping

## Abstract

**Background:**

The lack of access to mental health care could be addressed, in part, through the development of automated screening technologies for detecting the most common mental health disorders without the direct involvement of clinicians. Objective smartphone-collected data may contain sufficient information about individuals’ behaviors to infer their mental states and therefore screen for anxiety disorders and depression.

**Objective:**

The objective of this study is to compare how a single set of recognized and novel features, extracted from smartphone-collected data, can be used for predicting generalized anxiety disorder (GAD), social anxiety disorder (SAD), and depression.

**Methods:**

An Android app was designed, together with a centralized server system, to collect periodic measurements of objective smartphone data. The types of data included samples of ambient audio, GPS location, screen state, and light sensor data. Subjects were recruited into a 2-week observational study in which the app was run on their personal smartphones. The subjects also completed self-report severity measures of SAD, GAD, and depression. The participants were 112 Canadian adults from a nonclinical population. High-level features were extracted from the data of 84 participants, and predictive models of SAD, GAD, and depression were built and evaluated.

**Results:**

Models of SAD and depression achieved a significantly greater screening accuracy than uninformative models (area under the receiver operating characteristic means of 0.64, SD 0.13 and 0.72, SD 0.12, respectively), whereas models of GAD failed to be predictive. Investigation of the model coefficients revealed key features that were predictive of SAD and depression.

**Conclusions:**

We demonstrate the ability of a common set of features to act as predictors in the models of both SAD and depression. This suggests that the types of behaviors that can be inferred from smartphone-collected data are broad indicators of mental health, which can be used to study, assess, and track psychopathology simultaneously across multiple disorders and diagnostic boundaries.

## Introduction

### Background

Problems related to mental health are widespread, with 1 report estimating that 1 in 5 Canadians experience a problem related to mental health or addiction in any given year [1]. In 2018, 2.3 million Canadians went without adequate support for their mental health, with often-cited reasons including lack of information, time, or finances [2]. These impediments could potentially be addressed with the development and distribution of technology to aid in the diagnosis, tracking, and treatment of mental health disorders.

The widespread use of smartphones coupled with their sensing capabilities and persistent network connectivity makes them a promising platform for delivering these technologies. The sensors present on all modern smartphones offer a rich landscape of data that can be used to infer behavior or actions pertinent to an individual’s mental health state. These data could then serve as the basis for screening algorithms for mood and anxiety disorders such as depression, generalized anxiety disorder (GAD), and social anxiety disorder (SAD). The development of automated screening may be a crucial step in mental health care. Automated screeners could offer a low-cost monitoring system to individuals struggling with mental health problems and provide potentially useful information for practitioners of mental health care.

### Previous Work

A core aspect of this work is the extraction of relevant knowledge from the wide array of smartphone-sensed data. To convey a sense of scale of this class of data, consider that smartphones can sense, measure, and record geolocation, motion, ambient lighting conditions, ambient audio, communication patterns, and app use in real time. In a matter of weeks, tens of thousands of data points across these many data modalities can easily be collected from a single individual. Nonetheless, even with all these data, it remains quite challenging to build predictive models that are both accurate *and* transparent, in that they offer insight into what specific patterns of data, and what points in time, are driving models to make specific decisions or characterizations [3]. It is our goal with this work to address this challenge by building predictive models that are both accurate and transparent.

A general approach to this problem of knowledge extraction and interpretability has been to review the diagnostic criteria and the key symptoms of a mental health disorder and to look for data streams that would relate to these. In this approach, data are transformed into relevant knowledge by determining if the presence or absence of a symptom or characteristic of a disorder can be inferred from the available data. For example, key symptoms and criteria for depression include sleep disturbances (insomnia or hypersomnia) [4] and low energy [5]. The duration of sleep may be inferred by analyzing a combination of light and motion sensor data, screen activity, and ambient audio [6]. Although a person’s energy level is difficult to measure directly, proxy measures can be effectively created by observing the degree to which subjects leave their homes and travel throughout their environments [7] or by observing activity levels as measured by motion sensors [8].

This approach of using characteristics of disorder symptomology to drive the understanding of smartphone-collected data has been used in the modeling of SAD. Individuals with social anxiety are characterized by fear or avoidance of social interactions, ultimately stemming from the fear of negative evaluation from others [9]. Although the fear of evaluation is most certainly a latent construct, the avoidance of social situations can be objectively measured from smartphone-sensed data. One example of how this can be achieved is through the classification of different locations as inherently *social* or not, achievable using so-called *semantic location* data. Measuring the degree to which subjects travel to these inherently social locations (such as pubs and dancing venues) has been shown to be correlated with social anxiety levels [10]. Chow et al [11] demonstrated that greater time spent at home was correlated with stronger symptoms of social anxiety.

At the time of this writing, the authors are unaware of any studies that have used passively collected smartphone data to infer behaviors associated with GAD or to otherwise predict or screen for GAD.

With respect to depression, Ben-Zeev et al [12] demonstrated associations between the severity of depressive symptoms and smartphone-sensed geospatial activity, speech duration, and sleep duration. In a study that focused solely on GPS location–based data, Canzian and Musolesi [13] found associations between distance traveled and depressive symptoms. Increased time spent at home, as inferred from smartphone-collected GPS data, was also found to be associated with stronger symptoms of depression in studies by Saeb et al [7] and Farhan et al [14]. Both studies also found that a greater number of unique locations visited by subjects was associated with weaker symptoms of depression [7,14]. Studies by Wang et al [15] and Ben-Zeev et al [12] also used smartphone-recorded audio data to build a proxy measure for social interaction and found that this measure was negatively associated with depressive symptoms. Readers interested in a broader review of the associations between symptoms of mood disorders (including depression and additionally bipolar disorder) and smartphone-collected data are requested to refer the excellent review article by Rohani et al [16].

### Goal of This Study

This study aims to expand upon the existing body of work in using mobile sensing to predict certain aspects of mental health. Although the body of knowledge in this field continues to grow, much of it mainly focuses on mood disorders (depression and bipolar disorder), with much less of this work being undertaken in anxiety disorders. Studies have shown key features that act as predictors of depression, but many of these features have been motivated by general trends or associations with mental health that may extend beyond mood disorders to anxiety disorders. Furthermore, because anxiety disorders are commonly comorbid with depression [17], features that have been successful in predicting depression may also be successful in predicting anxiety disorders. The goal of this study is to propose a set of features, some novel and some existing, that have already been shown to be successful predictors of depression and to use this set of features in the independent prediction of GAD, SAD, and depression.

## Methods

### Recruitment and Demographics

The subjects were adults recruited from the general population using Prolific, a web-based recruitment platform [18]. Recruitment was conducted from July 2019 to December 2019. Subjects were not prescreened for psychiatric diagnoses. The study inclusion criteria were as follows: subjects must (1) reside in Canada, (2) be fluent in English, (3) own an Android phone, (4) have completed at least 95% of their previous Prolific studies successfully, and (5) have previously participated in at least 20 Prolific studies. The final criterion was used to ensure that subjects were proficient in using the Prolific system and were generally technology literate. There were no exclusion criteria for this study. Subjects were paid Can $18.50 (US $14.20) to participate in the study.

### Study Procedure

Subjects were entered into a 14-day observational study where a custom Android app was installed on their personal smartphone. The app collected both self-report measures of mental health (collected at the beginning and end of the study) and objective smartphone-sensed data, collected periodically and completely passively throughout the entire 2 weeks of the study.

### Self-report Measures and Mental Health Screening

Subjects completed 4 self-report measures, in digital form, within the study app, at the beginning and end of the 14-day study. A review by Belisario et al [19] found that self-administered survey scores did not differ significantly when deployed by the app compared with other delivery modes [19]. These self-reported surveys were completed on their own with no supervision by clinicians. Subjects completed the following four self-report measures of mental health: (1) the Liebowitz Social Anxiety Scale (LSAS), which is a 24-item self-report scale used in the assessment of SAD [20]; (2) the Generalized Anxiety Disorder 7-item (GAD-7) scale, which is a screening and assessment tool for GAD [21]; (3) the Patient Health Questionnaire 8-item (PHQ-8) scale, which is a screener and assessment tool for depression [22]; and (4) the Sheehan Disability Scale, which is a 3-item scale that assesses general impairment due to mental health [23]. Data from the Sheehan Disability Scale were not used in any of the analyses in this study.

Although all scales were completed at both the study intake and exit, only the values obtained at the exit were used in the analysis presented in this paper. This was done because the self-report measures asked respondents to evaluate symptoms over the *previous* 2 weeks; therefore, the window of symptom self-assessment at the study exit coincides with the window of electronic data collection. Changes with respect to baseline (ie, differences between scores measured at intake and exit) were not investigated because the goal was solely to determine whether digital technology could perform automated screenings from short-term data collection.

Exit values on the LSAS, GAD-7, and PHQ-8 were used to screen for SAD, GAD, and major depressive disorder (MDD), respectively, by applying thresholds to scores. A threshold of 60 was used with the LSAS scores to screen for SAD (generalized subtype), as recommended by Mennin et al [24]. This threshold was shown to screen for generalized social anxiety with sensitivity of 82% and specificity of 78% [25]. A threshold of 10 was used with the GAD-7 scores to screen for GAD. This threshold was shown to optimize sensitivity (89%) and specificity (82%) [21]. A threshold of 10 was used with the PHQ-8 scores to screen for depression, as recommended by Kroenke et al [22]. This threshold was shown to screen for major depression with sensitivity of 88% and specificity of 88% [22].

### Smartphone-Sensed Data Collection

The app collected data across 4 streams of data available on an Android smartphone: audio (from the microphone), geolocation (GPS sensor), screen state (screen on or off), and illuminance of the environment (light sensor).

Audio data were collected by using devices’ microphones to record the ambient audio of the environment for a 15-second duration, at a nominal rate of one 15-second recording every 5 minutes. To preserve privacy, these audio recordings were processed to yield 3 less invasive streams of data, after which the recordings were automatically destroyed. The first stream of audio-derived data is simply the average volume of each 15-second audio recording. The second stream of audio-derived data is a label for each audio recording, which indicates whether English-language speech was detected in the environmental audio at the time of each recording. Automatic speech recognition (ASR) software [26] was used to generate transcripts from each audio recording, and recordings that produced empty transcripts were labeled as containing no speech, whereas recordings that produced transcripts were labeled as containing speech. Finally, the third stream of audio-derived data is the entire set of ASR-detected English words. These words were stored in a randomized order, with no associated time information, to prevent the recreation of the original transcripts. Audio recordings were deleted by the software immediately after the extraction of mean volume, speech presence labels, and detected words.

The GPS location (latitude and longitude) was recorded at a nominal rate of once every 5 minutes. The state of the screen was not sampled periodically, but instead, the app recorded screen transitions (ie, turned off or on) whenever the state of the screen changed. Finally, the illuminance of the subjects’ environments, as measured by the devices’ light sensors, was recorded at a nominal rate of once every 10 minutes. We refer to these data sampling rates as nominal because, in practice, the observed frequencies are often lower than what was specified in the software because of the battery-preserving restriction imposed by the Android operating system in devices running Android version 6 and above [27].

### Feature Extraction

#### Feature Extraction Overview

Predictor variables, or *features*, were extracted from the raw smartphone-collected sensor data to act as inputs to the predictive models of SAD, GAD, and depression. These features are all hypothesized as capturing behaviors that are relevant to mental state, with the goal of using them as explanatory variables within the prediction models. The following sections describe each of the features that were used.

#### Daily Similarity

The ability to establish and maintain regularity in one’s patterns of activities has been associated with positive mental health [28,29]. Assuming that the volume of subjects’ environments could be interpreted as a proxy for activity, we developed a feature, called *daily similarity*, which quantifies how periodic the volume of the audio recordings was, with respect to a 24-hour or 1-day period. When the volume of the audio recordings was extracted and treated as time series data, there were clear peaks and troughs in the signal, corresponding to the daytime activity and nighttime inactivity, respectively. It was hypothesized that individuals with more regular patterns of activity and inactivity, as inferred from the peaks and troughs of this time series data, would have better mental health. The daily similarity feature was designed to compute a quantitative measure of the regularity, in time, of the sequence of these peaks and troughs—in other words, to what degree peaks and troughs occur at the same time across days.

**Figure 1:**
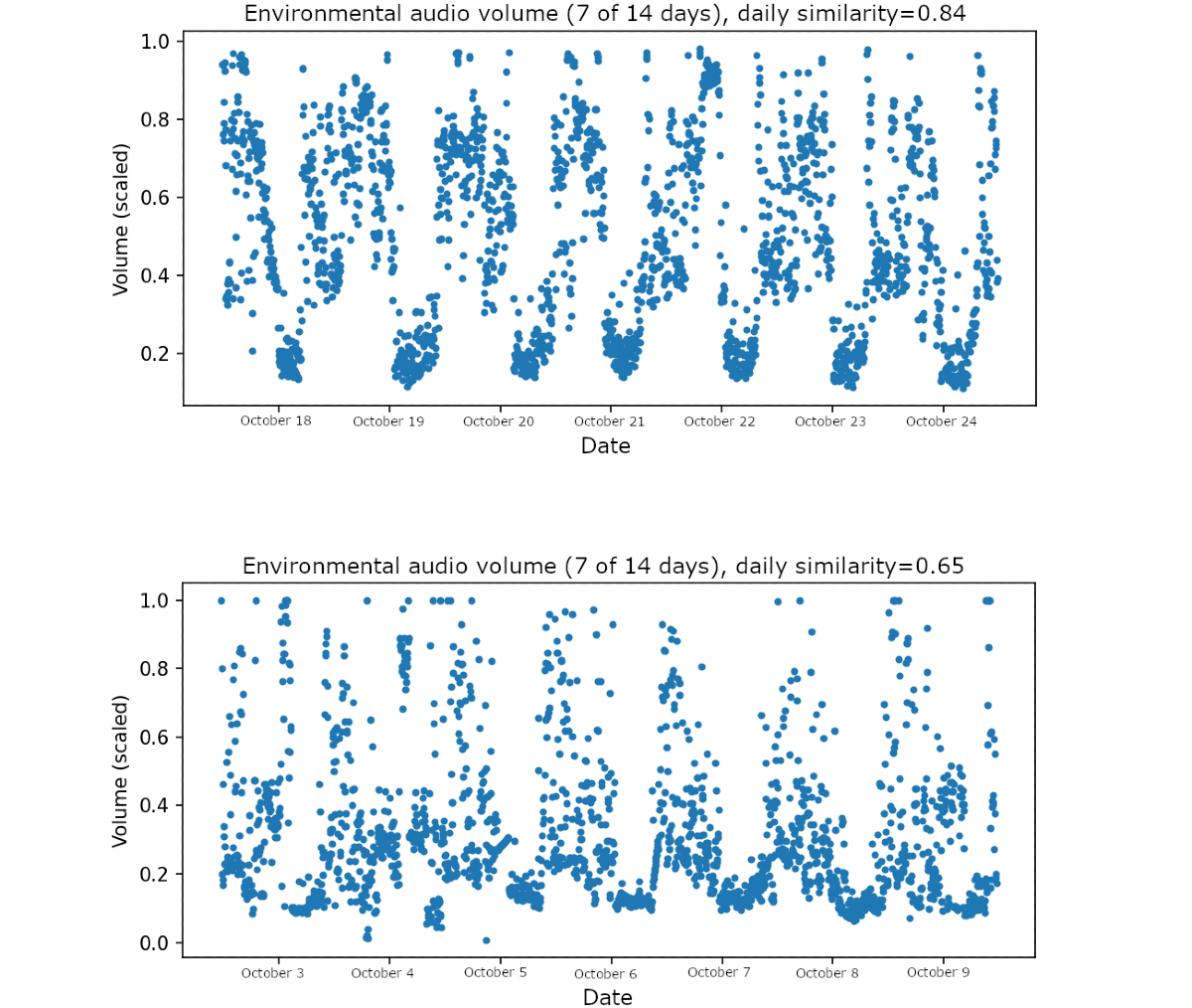
Environmental audio volume timeseries and values of the daily similarity feature for 2 study participants.

Mathematically, daily similarity is computed as the autocorrelation function of the volume time series of ambient audio recordings evaluated at a lag time of 24 hours. Figure 1 provides an illustration of 2 study participants’ volume time series data, with the computed daily similarity feature also annotated. Note that the first participant’s data, presented in the top half of the figure, is slightly more regular and has a higher daily similarity value than the participant whose data are presented in the bottom half of the figure. This feature was first presented and analyzed in a previous study by Di Matteo et al [30]. Note that the daily similarity feature, as computed in this work, only captures similarity on a short time scale; as a result, it is not clear if long-term stability in social rhythms would be captured.

#### Speech Presence

The avoidance of social interaction is a defining characteristic of SAD [31] and is also evident among individuals with depression [32]. The *speech presence* feature serves as a measure of social interaction by quantifying the presence of speaking voices in the periodic audio recordings. The speech presence feature is defined as the number of audio recordings labeled as containing speech divided by the total number of audio recordings. This feature was first proposed by Wang et al [15] and was confirmed to be a good correlate of anxiety and depression in our previous study [30]. We note that *any* speech in the environment, including the playback of recorded speech, will be detected as speech; therefore, this feature is not a perfect proxy for social interaction. The results of the study by Di Matteo et al [30] were reported under the same conditions.

#### Weeknight Sleep Disturbance

Sleep is also known to be a key factor in mental health disorders, with sleep disturbances being a common feature of anxiety and depression [4,33]. We compute a feature called *weeknight sleep disturbance,* which infers a proxy measure of sleep quality from the same audio volume time series data as the daily similarity feature. The underlying assumption of this feature is that when a subject’s environment is noisy during common hours of sleep, then it is likely that that the subject is either awake or not sleeping well. Specifically, this feature is a measure of the noisiness of a subject’s environment during common sleeping hours. The weeknight sleep disturbance feature is computed as the SD of the volume time series of ambient audio recordings for weeknights between midnight and 6 AM. Weekend evenings are excluded because some individuals may shift their sleep schedules on weekends, if they work weekdays and not weekends. The SD of the volume time series is used as a measure of noisiness, and not the volume itself, because noisy environments tend to produce volume recordings that are consistently chaotic (ie, high SD), yet the absolute volume of what can be considered *noisy* is often dependent on the microphone and further confounded by device-specific processing, including automatic gain control. This feature was first presented and analyzed in a previous study by Di Matteo et al [30].

#### Death-Related Words

In addition to the binary presence or absence of speech, the contents of that speech can be investigated to yield further features that may be related to the symptoms of anxiety and depression. The ASR-detected words produced from subjects’ audio recordings were analyzed using the Linguistic Inquiry and Word Count (LIWC) software tool [34] to generate a feature that captures relevant linguistic information. LIWC is a tool that can be used to characterize a text along numerous psychological dimensions or word categories. These categories are clusters of words that share a semantic meaning. For example, 1 category that can be explored with LIWC is *affect*, which comprises words relating to emotional states (some examples of the words belonging to the affect category are *happy* and *cried*). For each word category that is of interest, LIWC determines the percentage of words found in the text being analyzed which fall within the predefined dictionary of words for that category. We use LIWC to count the percentage of all words collected from the person that fall within LIWC’s *death* category and refer to this as the *death-related words* feature. In our previous work, we showed that the incidence of death-related words has been shown to have an association with symptoms of SAD, GAD, and depression [35]. The previous work of Di Matteo et al [35] also contains further methodological and implementation details of this LIWC-based feature extraction.

#### Number of Locations Visited

The number of locations that a person visits may also offer insight into mental state, as the avoidance behavior associated with SAD or the lack of energy associated with depression may result in an individual leaving the home less often than healthy individuals. To estimate the number of locations visited from the GPS location data, location data were first processed to identify the stationary points. A stationary point is defined as one in which a subject travels at a speed less than 1 km/h relative to the last recorded location point in time (assuming direct-line travel). This stationary location data were then processed using a clustering approach [36] to group closely located readings into distinct locations visited by the subject. The specific clustering algorithm used is called density-based spatial clustering of applications with noise [37], parameterized with an epsilon value of 150 m. The *number of locations visited* feature is simply the number of clusters produced when clustering a subject’s stationary GPS location data. This feature was adopted from the study by Saeb et al [7].

#### Number of Exits From the Home

The number of times a person leaves their home to visit another location is similarly hypothesized to be relevant to symptoms of anxiety and depression. Although subjects do not explicitly provide us with the location of their home, we infer their home location (or cluster) as the cluster in which participants spend the most amount of time between the hours of midnight and 6 AM, a method that has been used in previous studies [7,11]. Once the home cluster is identified, the *number of exits from the home* can be computed as the number of cluster transitions from the home cluster to any other nonhome cluster.

#### Screen Use

The *screen use* feature is computed as the proportion of time that each subject’s smartphone screen is on. It was computed from the screen on/off time series data. This feature was adopted from the study by Saeb et al [7].

#### Time in Darkness

The *time in darkness* feature is computed as the proportion of time that each subject’s light sensor readings measure an illuminance less than 5 lux, which corresponds to a very dark environment.

### Predictive Model Building and Evaluation

Our goal was to assess the capability of this set of 8 features to predict the screening results (ie, above or below a screening threshold) of SAD, GAD, and depression. To do so, logistic regression [38] models were built and independently evaluated for each of the 3 disorders. As a preprocessing step before model training, all 8 features were scaled to have a mean of 0 (SD 1). Any missing feature values (due to data loss) were imputed by median imputation [39]. Feature scaling and imputation were performed in an unbiased fashion, using only *training* data to estimate the mean, SD, and median to perform these operations.

To estimate the predictive performance, a cross-validation [40] scheme was used to provide an unbiased estimate of how well these models predict screening results for unseen subjects (ie, subjects whose data were not used to build or *train* the model). This method is often used in machine learning–based approaches. The cross-validation approach used was repeated *k*-fold cross-validation [41], with 5 folds and 20 repeats. In this approach, the data set is partitioned into 5 *folds* with approximately 17 subjects each. Then, four of those five folds were used to train a logistic regression model, and the accuracy was then evaluated by predicting the unseen screening results of the held-out fold. This process is repeated for a total of 20 times, where in each of the 20 iterations, the data set is split into a different random assignment of participant data to the 5 folds. As every repetition of the 5-fold cross-validation scheme produces 5 models to be trained and evaluated (1 for each of the held-out sets), a total of 100 logistic regression models were trained and evaluated for each of the 3 disorders. A repeated *k*-fold cross-validation scheme was used instead of simple cross-validation because the data set is relatively small and, therefore, the performance of the models can vary by chance depending on how the subjects are randomly assigned to folds.

The predictive performance of each of the 3 groups of models (GAD, SAD, and MDD) is reported as the mean (across the 100 models) of the area under the receiver operating characteristic (AUROC) [42], which is sometimes simply referred to as area under the curve. A modified version of a 1-tailed *t* test was used to assess whether the mean predictive performance of each group of models is significantly better than that of an uninformative model (which would produce random predictions). This corrected repeated *k*-fold cross-validation *t* test was developed by Bouckaert and Frank [43] to account for the fact that the performance of each model produced by a repeated *k*-fold cross-validation resampling is not independent, as models built using this strategy share training data.

Finally, to inspect and interpret the models and the relative influence of each feature within the model, a single model was trained using the entire data set for each of the 3 disorders under investigation. The coefficients of these *full* models will then be presented and discussed. These coefficients are x-standardized as all features were scaled to have mean of 0 (SD 1) before model building. This standardization was necessary because we wish to compare the effects of features that do not share a common unit of measurement or scale.

### Ethics and Privacy

All recruited subjects retained anonymity and were provided with randomized account log-ins for use in the study app. All data were transmitted to the study servers over encrypted channels and stored in an encrypted form. Audio recordings were processed entirely by automated software with no human intervention and were deleted immediately upon processing. The words contained in the transcripts of audio recordings were stored in a randomized order to prevent the recreation of speech, after which each transcript was destroyed. The study was approved by the University of Toronto Health Sciences Research Ethics Board (protocol #36687).

## Results

### Subject Demographics

Of the subjects who completed the study, 75% (84/112) yielded sufficient data for analysis.

Missing data were encountered because of the smartphone app; in some cases, sampling data were obtained at rates below the intended rate. For each participant and for every data stream (ie, audio, GPS, and light), the number of data samples collected in that stream was compared with the number of samples that would be collected given perfect periodic sampling at the desired sampling rate. If the number of samples was less than half of what would be expected, then features would not be computed from that data stream, and those associated features would be considered missing. Participants having four or more missing features were then considered to have insufficient data and were excluded from the analysis.

The gender balance of the study sample was 42% (35/84) female and 58% (49/84) male, with an average age of 28.8 (SD 8.6) years. The 84 subjects included in the analysis and the 28 subjects excluded from the analysis did not differ significantly in mean age, gender distribution, or mean score of any of the 4 self-report measures.

### Subject Mental Health Screening

Subjects were screened for SAD, GAD, and depression using the LSAS, GAD-7, and PHQ-8 instruments, respectively. The screening criteria and the number and percentage of positive screenings are summarized in Table 1. The prevalence of SAD, GAD, and MDD in this sample was higher than that in the general Canadian population [30]. We hypothesize that this may be, in part, explained by the fact that study subjects who rely upon Prolific or other crowdsourced work may be under precarious employment, which is linked to poor physical and mental health [44].

**Table 1:** Subject screening results for social anxiety disorder, generalized anxiety disorder, and depression (n=84).

Disorder	Screening criteria	Positive screenings, n (%)
Social anxiety disorder	LSAS^a^ score ≥60	32 (38)
Generalized anxiety disorder	GAD-7^b^ score ≥10	22 (26)
Major depressive disorder	PHQ-8^c^ score ≥10	31 (37)

^a^LSAS: Liebowitz Social Anxiety Scale.

^b^GAD-7: Generalized Anxiety Disorder 7-item.

^c^PHQ-8: Patient Health Questionnaire 8-item.

### Features Extracted From Smartphone-Collected Data

Summary statistics for all eight features extracted from the subjects’ smartphone-collected data are presented in Table 2. The daily similarity and weeknight sleep disturbance features do not lend themselves to intuitive interpretation, and we suggest referring to the mathematical definitions provided in the *Methods* section. The remainder of the features are quite simple and can be easily interpreted in the context of everyday behaviors. The speech presence feature has a mean of 0.15, which indicates that, on average, subjects spent 15% of their time (including sleeping time) in the presence of smartphone-detected speech. The mean value of the death-related words feature was 0.16%, indicating that these words were detected infrequently. The mean of the number of locations visited feature was 15, and the mean of the number of exits from the home feature was 15. The subjects’ devices had their screens in an on state 23% of the time, on average, and devices were in darkly lit environments 63% of the time.

**Table 2:** Summary statistics for all subjects’ features (n=84).

Feature	Value, mean (SD)	Value, minimum	Value, first quartile	Value, second quartile	Value, third quartile	Value, maximum
Daily similarity	0.80 (0.07)	0.45	0.77	0.83	0.85	0.90
Speech presence	0.15 (0.06)	0.01	0.11	0.15	0.20	0.30
Weeknight sleep disturbance	0.14 (0.06)	0.03	0.09	0.12	0.18	0.34
Death-related words	0.16 (0.10)	0.00	0.09	0.15	0.20	0.51
Locations visited	15 (9.90)	1	8	13	20.5	50
Exits from home	15 (6.90)	1	11	14	18.5	34
Screen use	0.23 (0.13)	0.02	0.12	0.21	0.29	0.60
Time in darkness	0.63 (0.14)	0.31	0.60	0.63	0.74	0.96

### Predictive Performance for Models of SAD, GAD, and MDD

Three independent logistic regression models were built and evaluated using all eight features to predict SAD, GAD, and depression. As noted in the *Methods* section, a repeated *k*-fold cross-validation scheme was used to acquire an unbiased estimate of the models’ predictive performance. The mean AUROC for each of the 3 disorder models is shown in Table 3. The SAD model achieved a mean AUROC of 0.64 (SD 0.13), the GAD model achieved a mean AUROC of 0.55 (SD 0.14), and the depression model achieved the best results with a mean AUROC of 0.72 (SD 0.12).

**Table 3:** Aggregate predictive performance of the resampled models of social anxiety disorder, generalized anxiety disorder, and depression.

Disorder model	AUROC^a^, mean (SD)	*t* test (*df*)	*P* value
SAD^b^	0.64 (0.13)	2.04 (99)	.02
GAD^c^	0.55 (0.14)	0.73 (99)	.23
MDD^d^	0.72 (0.12)	3.46 (99)	<.001

^a^AUROC: area under the receiver operating characteristic.

^b^SAD: social anxiety disorder.

^c^GAD: generalized anxiety disorder.

^d^MDD: major depressive disorder.

Table 3 also provides the results of a modified *t* test to compare whether the mean AUROC of each model was significantly better than that of an uninformative model (which performs with AUROC=0.5). Both the SAD model and the depression model performed significantly better than the uninformative model at a 5% significance level. The GAD model effectively performed on par with an uninformative model (mean AUROC 0.55), showing no effective capability to predict GAD.

### Feature Importance

**Figure 2:**
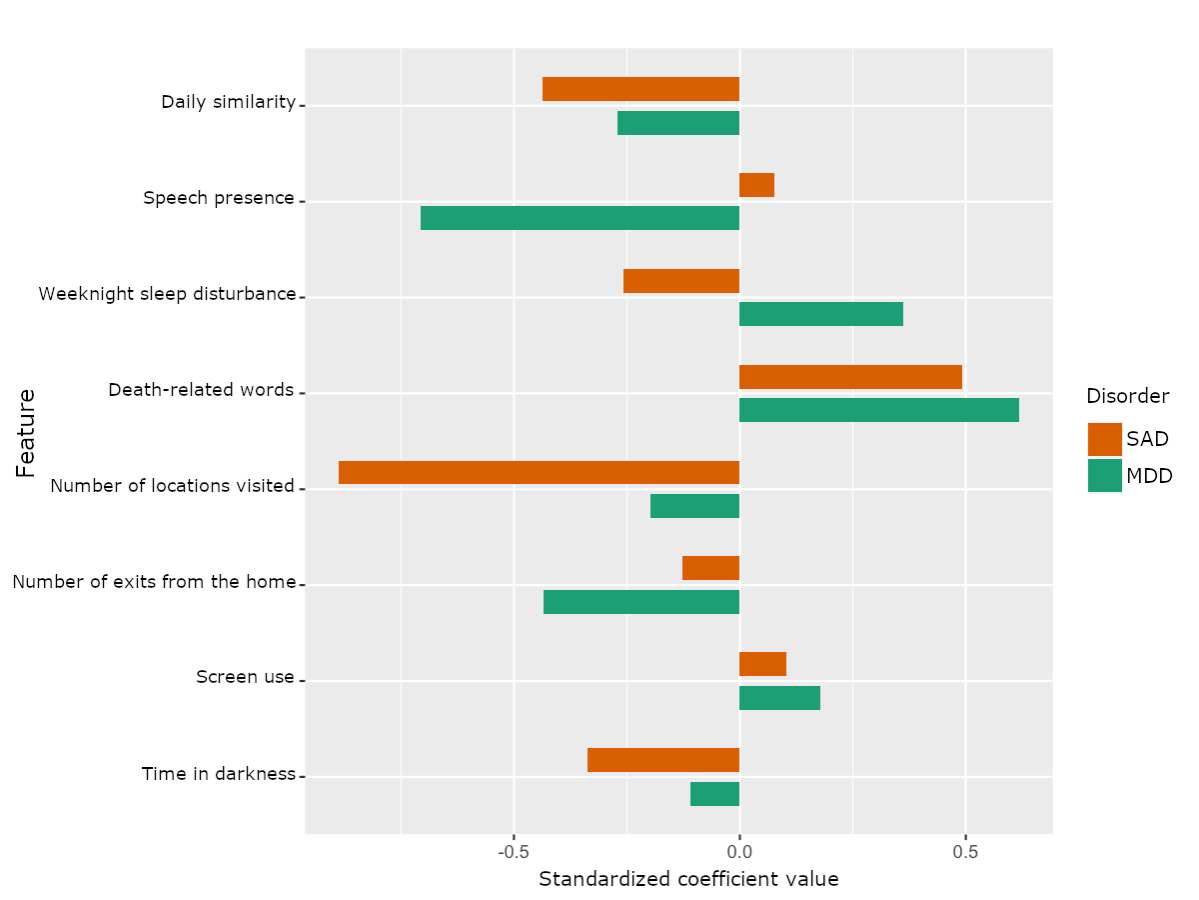
Comparison of logistic regression model coefficients by disorder. MDD: major depressive disorder; SAD: social anxiety disorder.

We sought to determine the relative importance of the features and to compare how feature importance may differ between the models for the 3 different disorders. The standardized logistic regression coefficients of the models of SAD and depression are shown in Figure 2. The GAD model was not included because it failed to achieve a significant predictive capability. As described in the *Methods* section, these coefficients come from a *single* model that was built on the entire data set of 84 subjects for each disorder, in contrast with the results in Table 3, for which many models’ performances were averaged (in the cross-validation scheme). The difference here is that the aim is not to measure performance but to inspect the model characteristics; hence, only 1 model per disorder, trained on the entire data set, is needed.

First, by looking at the signs of the feature coefficients, we can determine which features were found to decrease the odds of a positive screening, as negative coefficients correspond to a feature that is associated with *decreased* odds of a positive screening (a characteristic of the logistic regression modeling used). The following features were associated with decreased odds of a positive screen for both social anxiety and depression: daily similarity, number of locations visited, number of exits from the home, and time in darkness. This indicates that these features generally capture healthy behaviors with respect to social anxiety and depression. In contrast, the death-related words and screen use features were both associated with increased odds of positive screening for SAD and depression.

Two features, namely, speech presence and weeknight sleep disturbance, were found to have contrasting directionality with respect to screening for SAD and depression. Speech presence was associated with greatly *decreased* odds of a depression screening; however, speech presence was associated with a slight increase in the odds of a SAD screening. In addition, weeknight sleep disturbance was associated with increased odds of a depression screening but with decreased odds of a SAD screening.

Second, in terms of the relative magnitudes of their associated coefficients, several features stand out. For SAD, the daily similarity and number of locations visited features seem to be the strongest indicators of health, whereas the death-related words feature is the greatest risk factor for SAD. For depression, the speech presence and number of exits from the home features seem to be the strongest indicators of health, whereas weeknight sleep disturbance and death-related words features are the greatest risk factors for depression.

## Discussion

### Modeling and Feature Importance

The moderate success of the predictive models of SAD and depression is interesting when compared with the failure of the GAD model. This particular feature set seems unable to predict GAD, which may be related to how generalized anxiety manifests itself in subjects’ feelings and behaviors in a manner different from social anxiety or depression. Individuals with SAD commonly use behavioral avoidance to avoid situations that trigger their anxiety symptoms; they may choose specific jobs to avoid public speaking or avoid going to social gatherings [45]. The relative lack of or decrease in public speaking, traveling, and leaving the home are all physical behaviors that can be detected by some of the features used in this study. The speech presence feature can measure changes in the amount of speech, the number of locations visited feature can measure changes in the amount of travel, and the number of exits from the home feature directly measures how often one leaves the home. Although individuals with depression do not avoid specific situations for the same reasons that individuals with social anxiety do, depression is characterized by a general lack of motivation and energy [46]. This lack of motivation and energy also appears to manifest itself in behaviors (or lack thereof), which are detectible by our feature set (specifically the speech presence, number of locations visited, and number of exits from the home features).

This *behavioral* avoidance can be contrasted with the *cognitive* avoidance that is common in individuals with GAD [47], which includes maladaptive and somewhat pathological strategies such as distraction, worry, and thought suppression [48]. These are all strategies used by individuals with GAD to suppress their symptoms; however, they do not as easily manifest in the physical realm in a way that can be detected through, for example, GPS location data. In other words, it may be that this study’s feature set does a good job of detecting behaviors but a poor job in inferring the cognitive strategies that may often be used in a pathological manner to combat negative and, therefore, aversive feelings. Although it is true that screen use–based features could infer times when subjects were using distraction as a behavioral avoidance strategy, there are other possible explanations for screen time that might not include avoidance. The screen use data that we collected did not contain information on what apps were being used while the screen was on, which could be used to determine the motivation for using the phone. This more fine-grained use data that detailed which apps were in use (eg, games vs productivity apps such as email) would yield more insight into this type of avoidance and therefore may be more predictive of GAD.

A similar line of reasoning was presented in an earlier study of the audio-based features and how they were less strongly correlated with GAD symptom severity than the symptom severity of SAD and MDD [30]. It is worth noting that the additional features presented in this work (which were derived from GPS location, screen data, and light sensor data) also fail to effectively predict GAD.

### Comparing the Models of SAD and MDD

The predictive models of SAD and depression have similar performance, achieving mean AUROC values of 0.64 (SD 0.13) and 0.72 (SD 0.12), respectively. Numerous features appear to be of near-equal importance for both models, whereas some features are much more important for one of the two disorders (SAD and MDD). Comparing the 2 models, the proportion of death-related words detected in the environmental audio recordings was the greatest risk factor for both SAD and depression. The link between the use of death-related words and depression has been demonstrated in some previous studies [49,50], but we are not aware of any empirical studies that have demonstrated a link between death-related words and symptoms of SAD. Some researchers have proposed that death anxiety and fear of death may function as a transdiagnostic construct underpinning a range of mental disorders, including anxiety disorders [51]. If this hypothesis holds true, then the presence of death-related words in environmental audio may also serve as a proxy measure of death anxiety.

Continuing to compare the models of SAD and MDD, the daily similarity feature appears as a factor of good health with respect to both SAD and depression, in line with the hypotheses outlined in the description of this feature in the *Methods* section. That is, higher regularity in the subjects’ patterns of daily activities may be associated with better mental health.

A difference between the 2 models is that 2 features appear to act as predictors in opposite directions. First, the speech presence feature appears to be a critical indicator of health with respect to depression: subjects who spend more time in the presence of speech in their environment had greatly reduced odds of depression. The same is not true for SAD, in which the direction of this effect was reversed. Nonetheless, the value of the coefficient for speech presence in the SAD model is so low that it is considered an insignificant risk factor. The result that links more environmental speech to weaker symptoms of depression replicates results from previous studies [12,15]. It is interesting to note that more environmental speech was not associated with weaker symptoms of SAD. It may be the case that more information regarding whom the subject is speaking to (assuming that the speech is from present humans and not prerecorded speech from a device of some kind), and in what setting, is necessary to identify the key contexts in which social interactions are relevant to a subject’s symptoms of SAD.

The weeknight sleep disturbance feature is the second of 2 features that act in different directions within the models of SAD and depression. Sleep disturbance is a risk factor in the model of depression, a result that aligns with our original hypothesis. However, sleep disturbance was shown to decrease the odds of a positive screening of SAD. This indicates that sleep disturbance may be a positive factor with respect to social anxiety, if enhanced nighttime activity is indeed the result of social interaction.

Finally, there are 2 features that serve as positive factors for both SAD and depression, but with marked differences in magnitude. The number of locations visited feature is much more impactful in the model of SAD than in the model of depression, whereas the number of exits from the home feature is much more impactful for depression than for SAD. This may suggest that for depression, simply leaving the home and engaging in some form of activity is enough to preclude a positive screening. For SAD, simply leaving the home may not provide enough opportunity for social engagement to indicate a lack of social anxiety; however, visits to numerous locations (some of which may be social in nature) may suggest that an individual does not have SAD.

### Comparison With Other Studies

A number of studies have used smartphone-collected data in a similar fashion to build and evaluate predictive models of depression. Saeb et al [7] achieved a classification accuracy of 78.8% in detecting individuals with depression (which was defined as having a PHQ-9 score ≥5) using only location-based features. To enable more direct comparisons, 2 existing studies have also reported AUROC as their metric of accuracy in predicting depression using smartphone-collected data. Wang et al [52] reported an AUROC of 0.81 for a model that predicted depression using location, audio, and screen-based features. A study by Place et al [53] achieved an AUROC of 0.74 for detecting depressed mood using features derived from GPS location, audio, motion sensor data, phone and messaging metadata, screen data, and other device data.

Both the studies by Wang et al [52] and Place et al [53] differ from ours, however, in how they screen subjects for depression, as they used scores from the abbreviated 2-item PHQ-2 depression instrument. Furthermore, in both studies, participants could be considered to be drawn from a more homogenous sample, as they were both geolocated in particular metro areas. The participants in the study by Wang et al [52] were 48 undergraduate students at Dartmouth College, whereas the 73 participants in the study by Place et al [53] were all residents of the Boston area. The participants in our study were a mix of students and nonstudents and were geographically located across Canada in both rural and urban areas. The nature of the area in which subjects live and travel is of particular importance for location-based features, as individuals living in rural areas may exhibit different patterns of travel than those in urban areas.

Studies of anxiety disorders using smartphone-collected data are much less represented in the literature. A study by Boukechba et al [10] demonstrated strong correlations between smartphone-collected data and symptom severity of SAD, but no classification (ie, predictive screening) was performed. To our knowledge, there are currently no studies that have predicted or otherwise measured correlations between GAD and smartphone-collected data.

### Limitations

One limitation of this study is that the subjects’ state of SAD, GAD, and depression was determined using self-report measures as screeners. Although all the instruments used demonstrated acceptable accuracy for doing so, it is not clear how these results would compare had the diagnoses been performed using structured clinical interviews by trained clinicians.

Another limitation is related to the speech presence feature, which captures any intelligible speech within range of participants’ devices, including television or other media. More information about whom the subject is speaking with (ie, humans present in the environment, in comparison with recorded speech, be it from television, radio, etc) and where the individual is situated at the time of the recording may improve the ability to identify the key contexts in which social interactions are relevant to a subject’s symptoms of SAD.

Another set of key limitations involves the use of a particular smartphone system to collect data. First, all subjects were users of Android smartphones. Although our study excluded iOS users, 1 study of depression prediction from smartphone data that separately considered both iOS and Android users found similar results for both subgroups [14]. Second, the features derived from the smartphone-collected data in this study have some limitations. The speech presence feature and the death-related words features do not distinguish between speech produced by the subject and speech produced by other people. The screen use feature does not account for which app, if any, the subject is interacting with while the screen is on. Finally, the time in darkness feature does not distinguish between the device measuring low light conditions because the subject is in a dark room (eg, while sleeping) or the device simply being in a dark location (eg, in a pocket).

### Conclusions

This work contributes to the development of automated technology capable of screening individuals for SAD, GAD, and depression. To our knowledge, this is the first study to evaluate and compare how the same set of predictor variables derived from objective smartphone-collected data perform in predicting depression and 2 classes of anxiety disorders. This set of features was used to build models that had a significant capability in predicting SAD (mean AUROC 0.64, SD 0.13; *P*=.02) and depression (mean AUROC 0.72, SD 0.12; *P*<.001). Although the prediction of GAD was unsuccessful, we believe that we are the first to evaluate that disorder using this methodology and to propose key considerations that may yield success in predicting that disorder from smartphone-collected data.

## Abbreviations

ASR: automatic speech recognition
AUROC: area under the receiver operating characteristic
GAD-7: Generalized Anxiety Disorder 7-item
GAD: generalized anxiety disorder
LIWC: Linguistic Inquiry and Word Count
LSAS: Liebowitz Social Anxiety Scale
MDD: major depressive disorder
PHQ-8: Patient Health Questionnaire 8-item
SAD: social anxiety disorder
